# Supplementary material for: Use of a Taguchi Design in Hibiscus sabdariffa Extracts Encapsulated by Spray-Drying
Source: Foods. 2020 Jan 24;9(2):128. doi: 10.3390/foods9020128 (PMC7073635; doi:10.3390/foods9020128)
Supplement: Supplementary file 1 [file foods-09-00128-s001.pdf]

Supplementary materials

Use of a Taguchi Design in *Hibiscus sabdariffa* Extracts Encapsulated by Spray-Drying

Use of a Taguchi Design in *Hibiscus sabdariffa* Extracts Encapsulated by Spray-Drying

Migdalia Sarahy Navidad-Murrieta [1], Alejandro Pérez-Larios [2], Jorge Alberto Sanchéz-Burgos [1], Juan Arturo Ragazzo-Sánchez [1], Gabriel Luna-Bárcenas [3] and Sonia G. Sáyago-Ayerdi [1,*]

1 Tecnológico Nacional de México/Instituto Tecnológico de Tepic, Av. Tecnológico 2595, Lagos del Country, Tepic 63175, Nayarit, México; murrieta_26@hotmail.com (M.S.N.-M.); jragazzo@ittepic.edu.mx (J.A.S.-B.) jsanchezb@ittepic.edu.mx (J.A.R.-S.)

2 División de Ciencias Agropecuarias e Ingenierías, Centro Universitario de los Altos/Universidad de Guadalajara, Av. Rafael Casillas Aceves 1200, Tepatitlán de Morelos 47600, Jalisco, México; alarios@cualtos.udg.mx

3 Centro de Investigación y Estudios Avanzados (CINVESTAV) del Instituto Politécnico Nacional, Unidad Querétaro, Libramiento Norponiente 2000, Fracc. Real de Juriquilla, Santiago de Querétaro 76230, Qro., México; gabriel.luna@cinvestav.mx

* Correspondence: ssayago@ittepic.edu.mx; Tel.: +52-311-211-94-00 ext. 234

Received: 11 November 2019; Accepted: 13 January 2020; Published: 24 January 2020

Table S1. Outlet temperate in treatments of *Hibiscus* extracts (*Hibiscus sabdariffa*) spray-drying with different proportions of MD and GA[1,2].

Treatment	Inlet temperature (°C)
T_1	65 ± 0.5
T_2	64 ± 0.5
T_3	64 ± 0.5
T_4	93 ± 0.5
T_5	92 ± 0.5
T_6	95 ± 0.5
T_7	64 ± 0.5
T_8	84 ± 1.5

[1] 1 Values are the mean (n = 3). [2] MD: Maltodextrin DE 10, GA: Gum arabic.

Table S2. ANOVA general linear model results showing p-values of each factor investigated from the Taguchi L8 design on the selected responses variables.

Variable	SS[1]	Df[2]	MS[3]	F[4]	p
**FST Content**					
{1} Solvent of extraction	1155.422	1	1155.422	19320.26	0.000000
{4} Extract:carriers ratio (w/w)	91.270	1	91.270	1526.17	0.000000
{6} Homogenization	11.569	1	11.569	193.45	0.000000
{5} Carriers ratio (MD + GA)[5]	10.970	1	10.970	183.43	0.000000
{2} Decoction (°C/min)	1.511	1	1.511	25.27	0.000124
{7} Inlet temperatura (°C)	0.673	1	0.673	11.26	0.004020
{3} *Hibiscus* concentration	0.061	1	0.061	1.02	0.326443
Residual	0.957	16	0.060		

Table S2. *Cont.*

**AOX**					
<u>ABTS</u>					
{1} Solvent of extraction	2414.040	1	2414.040	26792.05	0.000000
{2} Decoction (°C/min)	56.339	1	56.339	625.28	0.000000
{3} *Hibiscus* concentration	62.708	1	62.708	695.96	0.000000
{4} Extract:carriers ratio (w/w)	128.787	1	128.787	1429.33	0.000000
{5} Carriers ratio (MD + GA)	15.000	1	15.000	166.47	0.000000
{6} Homogenization	76.048	1	76.048	844.01	0.000000
{7} Inlet temperatura (°C)	46.757	1	46.757	518.93	0.000000
Residual	1.442	16	0.090		
<u>DPPH</u>					
{1} Solvent of extraction	1322.908	1	1322.908	3359.843	0.000000
{4} Extract:carriers ratio (w/w)	22.469	1	22.469	57.065	0.000001
{2} Decoction (°C/min)	20.698	1	20.698	52.568	0.000002
{6} Homogenization	8.170	1	8.170	20.749	0.000324
{3} *Hibiscus* concentration	0.833	1	0.833	2.116	0.165080
{5} Carriers ratio (MD + GA)	0.738	1	0.738	1.874	0.189882
{7} Inlet temperatura (°C)	0.306	1	0.306	0.776	0.391414
Residual	6.300	16	0.394		
<u>FRAP</u>					
{1} Solvent of extraction	763.3372	1	763.3372	70317.90	0.000000
{2} Decoction (°C/min)	4.3148	1	4.3148	397.48	0.000000
{3} *Hibiscus* concentration	3.4680	1	3.4680	319.47	0.000000
{4} Extract:carriers ratio (w/w)	51.4172	1	51.4172	4736.51	0.000000
{5} Carriers ratio (MD + GA)	39.7565	1	39.7565	3662.34	0.000000
{6} Homogenization	17.2442	1	17.2442	1588.52	0.000000
{7} Inlet temperatura (°C)	54.2813	1	54.2813	5000.35	0.000000
Residual	0.1737	16	0.0109		

[1] SS. Sum of Squares
[2] df. Degree of Freedom
[3] MS. Mean Square
[4] F. F- Value
[5] MD: Maltodextrin DE 10, GA: Gum arabic.

Figure S1. Taguchi analysis for main effects: a) FST Content, AOX b) ABTS, c) DPPH and d) FRAP, ordinate values are the averaged one for each factor and abscissa shows the difference between highest and lowest response obtained for each independent factor

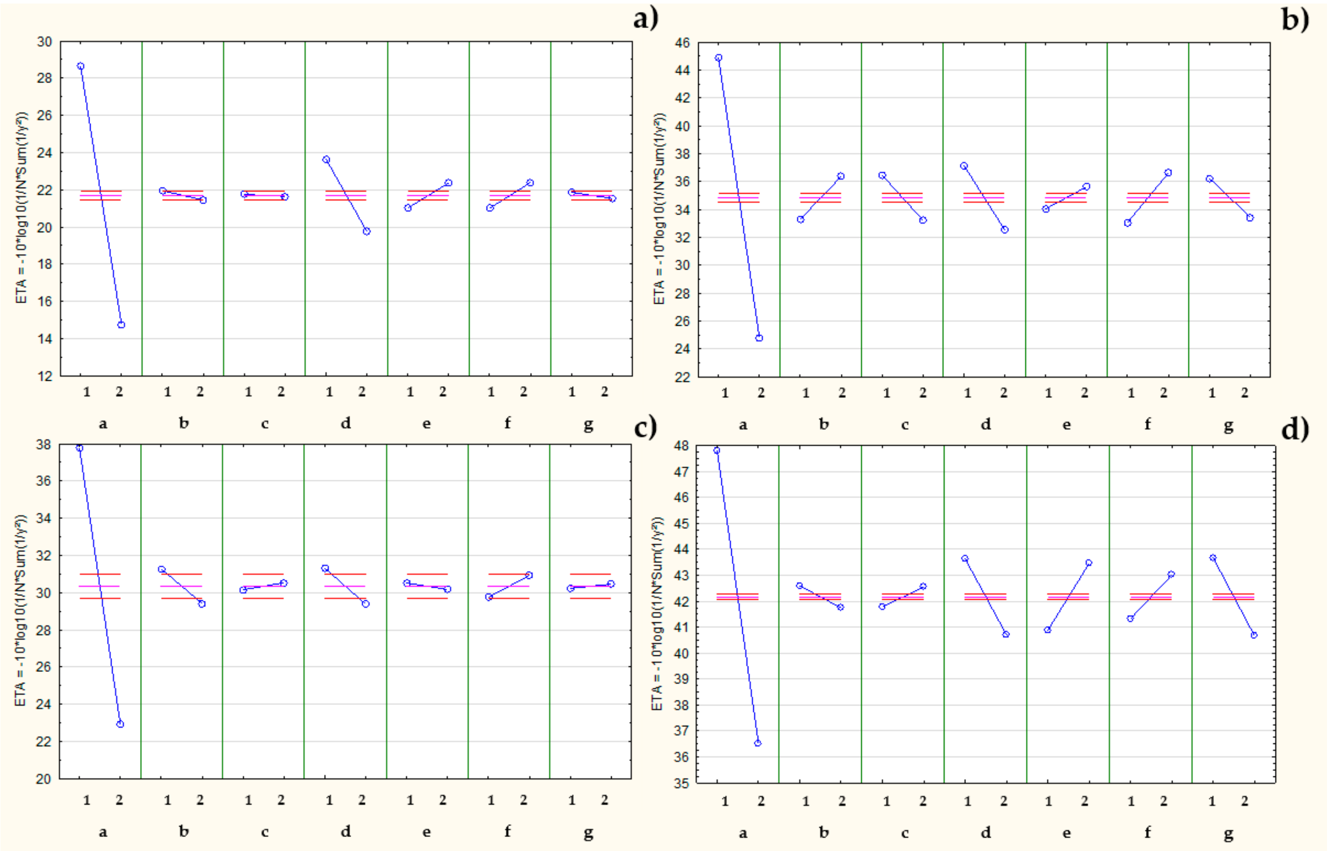

Abscissa values:

Level	Solvent of extraction (a)	Decoction (ºC/min) (b)	*Hibiscus* concentration (%) (c)	Extract:carriers ratio (w/w) (d)	Carriers ratio (MD + GA)[1] (%) (e)	Homogenization (rpm) (f)	Inlet temperature (°C) (g)
**1**	Water	NA[2]	1	1:1	80:20	5,000	110
**2**	Ethanol 20%	100 / 5	2.5	1:2	90:10	10,000	150

1. MD: Maltodextrin DE 10, GA: Gum arabic
2. NA
